# Effect of Water on Survival and Development of Diapausing Eggs of *Apolygus lucorum* (Hemiptera: Miridae)

**DOI:** 10.1371/journal.pone.0161657

**Published:** 2016-09-07

**Authors:** Yinli Jin, Peiyu Chen, Yanfang Zhang, Guo Tian, Hongqiang Feng, Youming Hou

**Affiliations:** 1 Fujian Province Key Laboratory of Insect Ecology, Department of Plant Protection, Fujian Agriculture and Forestry University, Fuzhou, 350002, Fujian, China; 2 Henan Key Laboratory of Crop Pest Control, Key Laboratory of Integrated Pest Management on Crops in Southern Region of North China, International Joint Research Laboratory for Crop Protection of Henan, Biological Pesticides Engineering Research Center of Henan Province, Institute of Plant Protection, Henan Academy of Agricultural Sciences, Zhengzhou, 450002, Henan, China; 3 Nanyang Academy of Agricultural Sciences, Nanyang, 473008, Henan, China; USDA Agricultural Research Service, UNITED STATES

## Abstract

The green mirid bug *Apolygus lucorum* is a regional pest of multiple crops in northern China, and the survival and development of diapausing eggs during winter plays an important role in the population dynamics of this species. The effect of water on the survival and development of *A*. *lucorum* eggs was investigated using laboratory-induced diapause. Diapausing eggs were exposed to various humidity regimes under three conditions: (1) termination of diapause with exposure to warm long-day (WLD) conditions (i.e., 26 ± 1°C and 75 ± 5% relative humidity (RH) under a photoperiod of 16 hours light and 8 hours dark), (2) termination of diapause by chilling at 4°C, or (3) during the post-diapause stage, i.e., from transfer to WLD conditions after chilling, until the hatching of nymphs. The results indicate that water availability is crucial for the post-diapause resumption of development of *A*. *lucorum*. However, exposure to excessive moisture was detrimental, as indicated by a decrease in diapause termination rate and a prolonged pre-hatching period of diapausing eggs, compared to limited moisture conditions. This implies that both too dry and too humid environmental conditions would suppress survival and postpone hatching of overwintered *A*. *lucorum* eggs, and might explain why this pest has not caused severe damage in either southern or western China where the respective climates are very humid or dry.

## Introduction

Commercialization of transgenic Bt (*Bacillus thuringiensis*) cotton has successfully controlled the cotton bollworm, *Helicoverpa armigera* (Hübner) in northern China, and caused a sharp decline in the use of broad-spectrum insecticides. However, mirid bugs have taken the place of *H*. *armigera* in this region of China and become widespread pests on Bt cotton and other crops [1–4]. The green mirid bug *Apolygus lucorum* (Meyer-Dür) (Hemiptera: Miridae) is currently the dominant species on Bt cotton, jujube tree *Ziziphus jujuba* Mill., grape tree *Vitis vinifera* L. and other crops in northern China [5–7]. This pest overwinters as diapausing eggs laid inside tissues of host plants, predominantly jujube trees, grape trees and weeds adjacent to cotton fields [6–8]. In the spring, *A*. *lucorum* produces 1 or 2 generations on herbaceous weeds or fruit trees and moves to cotton fields in mid-to-late June to produce 2 generations there [5]. Survival and development of the overwintering generation plays a key role in the life cycle of this species, and the increasing acreage of fruit trees [9] has provided more overwintering sites and has likely contributed to the increased regional pest status of *A*. *lucorum* [5, 8]. Thus, understanding the survival and development of diapausing eggs, and how environmental factors influence the hatching process, is critical for accurate forecasting of the population dynamics of this pest and for providing guidance for control practices.

In orchards, *A*. *lucorum* lays diapausing eggs in winter buds, pruning wounds and stubs, or beneath the bark of grape and jujube trees [6–8]. Each spring the newly hatched nymphs feed directly on tender buds, leaves and subsequent flowers of jujube and grape trees causing considerable loses, and this has attracted much research interest in recent years [6–8, 10]. Nymphs hatch from overwintered eggs synchronously with bud sprouting of fruit trees if the eggs are embedded in sprouting buds [6, 7]. However, the hatching may be delaying and the hatching rate may be strongly affected by rainfall if the eggs are embedded in dormant buds or dead plant parts (e.g. dead buds, bark and pruned stubs) [6, 7, 10]. This difference might be attributable to whether the egg can absorb water from environment or not. The eggs embedded in sprouting buds can absorb water from living and actively growing plant tissues, but eggs cannot absorb any water if the plant tissues are dead or dormant. Such eggs cannot develop until they obtain enough water from rain [6, 10].

It is clear that the overwintered *A*. *lucorum* eggs embedded in dead plant tissues cannot hatch without rainfall, natural or simulated via soaking [10]. Despite the evidence supporting the crucial role played by water in the survival of overwintered *A*. *lucorum* eggs, details of how water influences diapause development, and of which phase of diapause is sensitive to water or drought, are still absent. Rather than using field-collected eggs of unclear developmental stage [6, 7, 10], the present study used laboratory-induced diapausing and post-diapause eggs to elucidate which stage of diapause is most sensitive to water and to determine the relationship between egg development and moisture.

## Materials and Methods

### Insects and Collection of Diapausing Eggs

The laboratory population of *A*. *lucorum* was established originally by collecting nymphs and adults from weeds and cotton in Huaiyang County (33° 44′ N, 114° 53′ E), Henan Province, China, in July 2007. The laboratory culture was augmented by further adults from the same place in 2009. They were reared successively with fresh green bean pods (*Phaseolus vulgaris*) under warm long-day (WLD) conditions, i.e., at 26 ± 1°C and 75 ± 5% relative humidity (RH) under a photoperiod of 16 hours light and 8 hours dark (16L:8D) [11, 12]. Eggs from the cultures were collected and incubated within wet filter paper instead of bean pods, as described by Feng et al. [12, 13]. Automatically controlled environmental chambers (model PQX-280A-3H, Ningbo Laifu Technology Co., LTD, Ningbo, Zhejiang, China) were used to provide the desired conditions during insect rearing and for all the experiments described below. The light intensity in the rearing chambers was about 3000 lux provided by eight fluorescent tubes installed vertically at the left and right sides of each chamber.

**Fig 1:**
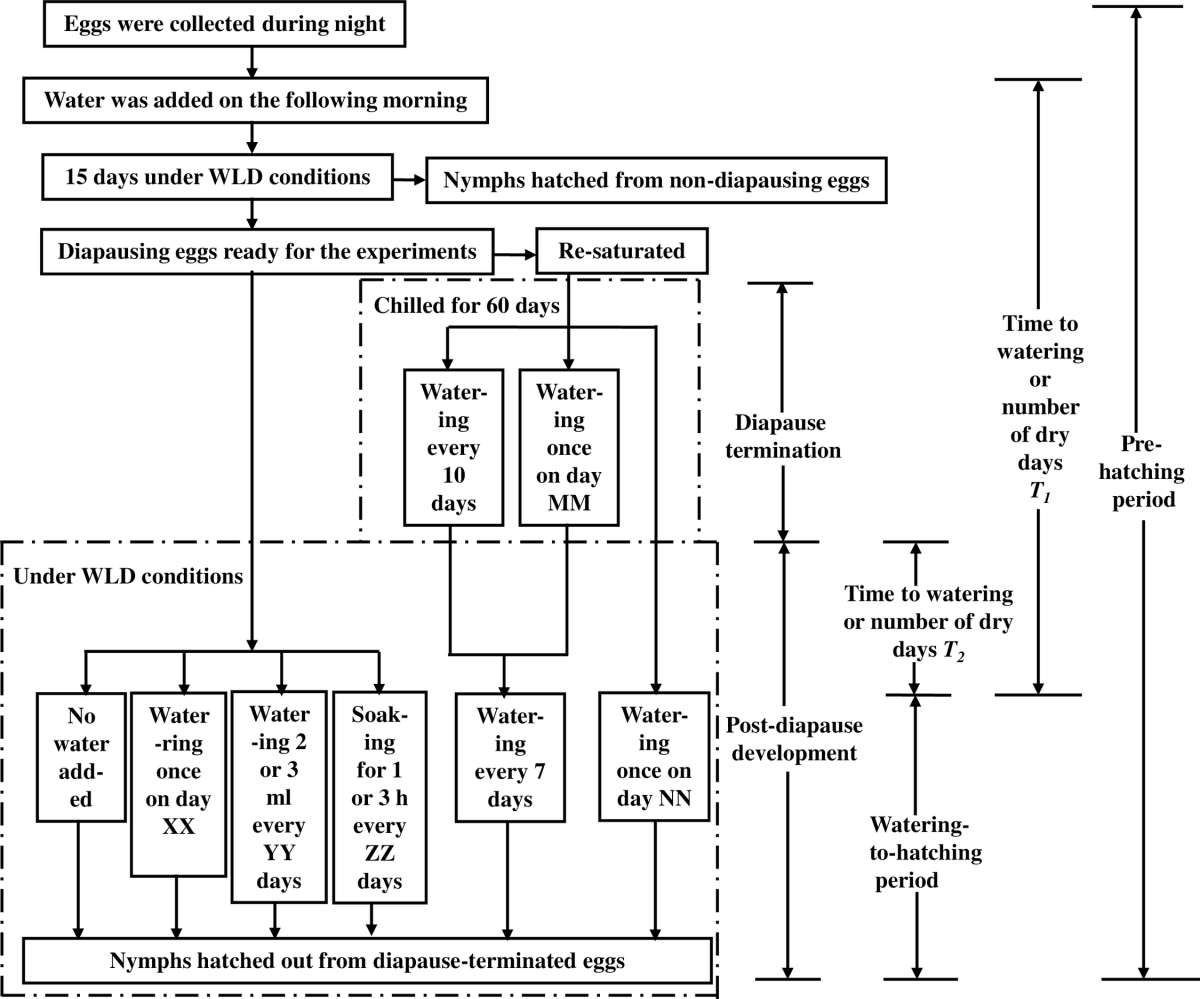
Flow chart showing water treatments during the diapause/post-diapause stage under warm long-day conditions, during the diapause termination (chilling) stage, and during the post-diapause stage (from transfer to WLD conditions after chilling until the hatching of nymphs). Watering once on day XX denotes watering on day 24, 35, 53, 63, 76, 84, 95, or 109 of incubation, respectively. Watering 2 or 3 ml every YY days means watering with either 2 or 3 ml water every 3 (watering with only 2 ml), 5, 10, 15, 20 and 30 days, respectively. Soaking for 1 or 3 h every ZZ days means soaking eggs for either 1 or 3 h every 5, 10 and 15 days, respectively. Watering once on day MM denotes watering once on day 0, 10, 20, 30, 40 or 50 of chilling, respectively. Watering once on day NN denotes watering once on day 0, 4, 6, 8, 10, 12, 14, 16, 18, 20 or 22 after transfer to WLD conditions, respectively.

A total of 2000 newly-hatched nymphs from the laboratory colony were reared under a short-day (8L:16D) photoperiod at 26 ± 1°C, 75 ± 5% RH until the adult females were ready to oviposit. The eggs produced under the above conditions were mainly diapausing eggs [13]. Eggs were collected during the night by placing uncovered Petri dishes with filter paper into the rearing box. Each 90 mm-dia. plastic Petri dish was lined with four layers of water-saturated filter paper disks of 70 mm diameter [13]. The filter paper with eggs dried out and were re-saturated the next morning with about 3 ml of distilled water, then the Petri dishes were covered and incubated under WLD conditions for about 15 days to sort out the diapausing eggs from the non-diapausing ones according to the criteria described by Feng et al. [13] and Chen et al. [14] (Fig 1). In brief, the non-diapausing eggs turn pale yellow and the red eyes of the embryos can be seen through the egg shell at the 5^th^ day of incubation, and nymphs hatch from such eggs within 15 days under WLD conditions (in fact, most nymphs hatched from non-diapausing eggs in about 8 days). Diapausing eggs, on the other hand, turn orange yellow without the appearance of red eyes at the 5^th^ day of incubation. Therefore, these morphological differences were suggested as criteria for the separation of diapausing and non-diapausing eggs. No supplementary water was added during the 15-day incubation and the filter paper with diapausing eggs generally dried out after about 10 days of incubation. These diapausing eggs were then used for the following experiments (Fig 1).

### The Determination of Developmental Stages of Diapause Eggs

Insect diapause is considered as a dynamic process consisting of 3 successive phases, i.e., pre-diapause, diapause and post-diapause, and the diapause can be subdivided into initiation, maintenance and termination phases [15]. The *A*. *lucorum* eggs enter diapause within 24 hours after oviposition as indicated by the pause in embryo development [16], but chilling the diapausing eggs at low temperatures of 2–4°C for about 60 days can terminate egg diapause successfully [17, 18]. Thus in this study, on a provisional basis, we regard the stage from oviposition to the end of the chilling treatment as the diapause stage (including diapause initiation, maintenance and termination), and we regard the stage from transfer to WLD conditions after chilling until the hatching of nymphs as the post-diapause stage (Fig 1). Exposing the diapausing eggs to constant light at high temperature also can terminate diapause [17] and in our pilot experiment the diapausing eggs hatched when kept under WLD conditions for a long time. In this situation, we could not separate the diapause and post-diapause stages clearly, and we refer to this as the ‘diapause/post-diapause’ stage in the present paper (Fig 1). Hereafter, the time duration from oviposition to hatching of nymphs is referred to as the pre-hatching period, and the duration from the transfer to WLD conditions after chilling until the hatching of nymphs is referred to as the post-diapause development duration. In addition, within this post-diapause development duration, the period from watering to hatching of nymphs is referred to as “the watering-to-hatching period” (Fig 1).

### Water Treatments during the Diapause/Post-Diapause Stage under WLD Conditions

Diapausing eggs embedded in filter paper placed in plastic Petri dishes were kept under constant WLD conditions for hatching, and monitored daily for about 4 months. Under WLD conditions, filter papers with eggs in a covered Petri dish generally dry out about 6 days after watering with 2 ml of distilled water. Therefore, watering with 2 ml of distilled water every 5 days (Table 1, Treatment K) produced a “limited moisture” condition, while more frequent watering or adding larger volumes of water at a time (Table 1, Treatments J, P, U and X, including watering with 2 ml of distilled water every 3 days, 3 ml of distilled water every 5 days and soaking 1 h or 3 h every 5 days, respectively) produced an “excessive moisture” condition. Other less frequent watering produced a “limited dry” condition (Table 1). No water added from beginning to end produced a “dry” condition (Table 1, Treatment A). On this basis, water was supplied in the following ways during the incubation of diapausing eggs under WLD conditions (Table 1, Fig 1):

1. no water added—the diapausing eggs embedded in the filter paper were placed in covered plastic Petri dishes and kept dry (Table 1, Treatment A);
2. watering once by spraying 2 ml distilled water for each Petri dish on day 24, 35, 53, 63, 76, 84, 95, or 109 of incubation, respectively (Table 1, Treatments B, C, D, E, F, G, H and I, respectively);
3. watering more than once by spraying 2 ml distilled water for each Petri dish every 3, 5, 10, 15, 20 and 30 days, respectively, and allowing to dry naturally (Table 1, Treatments J, K, L, M, N and O, respectively) (according to our pilot experiments, 2 ml of distilled water could be absorbed completely by the four layers of dry filter paper (70 mm in diameter) placed in the Petri dishes);
4. watering more than once by spraying 3 ml distilled water for each Petri dish every 5, 10, 15, 20 and 30 days, respectively, and allowing to dry naturally (Table 1, Treatments P, Q, R, S and T, respectively). For the 3 ml treatments, there was an obvious excess of water, and it did not run off when the Petri dishes were inverted;
5. soaking eggs in distilled water for either 1 or 3 h every 5, 10 and 15 days, respectively (Table 1, Treatments U, V, W, X, Y and Z, respectively).

Between 105 and 551 diapausing eggs were used in each treatment (Table 1).

**Table 1:** Diapause termination rate and average pre-hatching period for diapausing eggs of *A*. *lucorum* exposed to different water treatments under warm long day conditions.

Water treatment				Number of eggs tested	Diapause termination rate (%)	Average pre-hatching period (days) ± S. E.*
Watering regime	Amount or duration	Time or frequency	Treatment			
No water supply	0 ml	NA	A^††††^	360	0.55 a	118.00 k**
Watering once	2 ml	on day 24	B^†††^	271	89.38 fgh	78.64 ± 11.47 bc
		on day 35	C^†††^	551	86.98 ef	84.18 ± 11.87 de
		on day 53	D^†††^	105	84.11 cdef	85.40 ± 10.83 efg
		on day 63	E^†††^	180	80.77 cd	87.92 ± 8.78 h
		on day 76	F^†††^	358	91.39 gh	93.36 ± 5.03 i
		on day 84	G^†††^	164	85.54 def	99.22 ± 4.11 j
		on day 95	H^†††^	293	71.86 b	106.20 ± 1.40 k
		on day 109	I^†††^	336	0.89 a	123.00 ± 0.00 k***
Watering more than once	2 ml	every 3 days	J^††^	306	88.31 efg	84.56 ± 12.51 de
		every 5 days	K^†^	180	91.21 gh	72.76 ± 10.62 a
		every 10 days	L^†††^	158	77.50 bc	77.06 ± 10.92 b
		every 15 days	M^†††^	339	78.89 c	87.51 ± 10.77 h
		every 20 days	N^†††^	334	89.58 fgh	84.87 ± 9.91 ef
		every 30 days	O^†††^	302	92.43 hi	87.45 ± 11.39 h
	3 ml	every 5 days	P^††^	146	83.11 cde	76.38 ± 10.72 b
		every 10 days	Q^†††^	118	94.17 hij	77.87 ± 11.04 b
		every 15 days	R^†††^	361	90.91 gh	85.54 ± 12.32 fg
		every 20 days	S^†††^	122	97.58 j	81.78 ± 12.34 d
		every 30 days	T^†††^	142	78.47 bc	85.64 ± 11.58 fg
Soaking	1 h	every 5 days	U^††^	145	82.99 cde	84.36 ± 12.01 de
		every 10 days	V^†††^	142	84.72 cdef	85.83 ± 14.32 fg
		every 15 days	W^†††^	156	79.11 cd	87.40 ± 14.36 gh
	3 h	every 5 days	X^††^	159	85.71 def	80.00 ± 13.25 c
		every 10 days	Y^†††^	198	92.00 ghi	82.26 ± 11.82 d
		every 15 days	Z^†††^	237	94.98 ij	84.97 ± 11.35 efg

* Values followed by the same lowercase letter are not significantly different within each column (P < 0.05).

** Only one nymph hatched.

*** Only two nymphs hatched.

^†^ “limited moisture” condition.

^††^ “excessive moisture” condition.

^†††^ “limited dry” condition.

^††††^ “dry” condition.

### Water Treatments during the Diapause Termination (Chilling) Stage

Disks of filter papers with diapausing eggs were re-saturated with about 3 ml of distilled water for each plastic Petri dish and were subjected to chilling at 4°C under dark conditions (photoperiod of 0L:24D) in a refrigerator for 60 days. During chilling, the diapausing eggs were supplied with water via of seven regimens, which included spraying approximately 2 ml of distilled water every 10 days, spraying only once at the beginning of chilling (i.e., day 0) or spraying at 10, 20, 30, 40 or 50 days post-chilling initiation (Fig 1). While supplying water, the Petri dish was taken out of the refrigerator and exposed to light for less than 30 s. At 4°C, watering with 2 ml of distilled water every 10 days produced “excessive moisture” while other treatments produced the “limited moisture” or “dry” condition. After 60 days of chilling, diapause-terminated eggs were transferred to WLD conditions for hatching, and the incidence of hatching was monitored daily for about 40 days (Fig 1). Water was added once a week to keep the filter paper moist during hatching. Between 190 and 473 diapausing eggs were used for each treatment.

### Water Treatments during the Post-Diapause Stage

Diapausing eggs were chilled at 4°C and maintained in darkness for 60 days. Disks of filter paper with diapausing eggs were re-saturated with about 3 ml of distilled water only once before chilling. After chilling, the eggs were transferred to WLD conditions for post-diapause development. Then, the eggs were sprayed with 2 ml of distilled water only once either at the day of transfer (i.e., day 0) or at 4, 6, 8, 10, 12, 14, 16, 18, 20 or 22 days after transfer, respectively (Fig 1). This resulted in 0, 4, 6, 8, 10, 12, 14, 16, 18, 20 or 22 days of “dry” conditions. The incidence of hatching was monitored daily for about 40 days (Fig 1). Between 52 and 211 diapausing eggs were used successfully for each treatment.

### Statistical Analysis

Egg diapause termination rates were calculated and compared using Bayesian methods [19]. The differences in the pre-hatching period, the post-diapause development duration and the watering-to-hatching period were analyzed with Kruskal-Wallis tests and multiple comparisons were performed with Dunn’s test [20]. In the comparison, the development duration of non-diapause eggs, i.e., eggs of populations consecutively reared under WLD conditions, was used as a control. Nonlinear regression models were used for analysis in this paper, of which, the relationship between the pre-hatching period and the time to watering (number of dry days experienced before eggs were watered during the pre-hatching period) (*T*_*1*_) was fitted with an exponential regression, the relationship between post-diapause development duration of eggs and the time to watering (number of dry days experienced before eggs were watered during the post-diapause stage) (*T*_*2*_) was fitted with a logistic regression [21]. All statistics were performed with R packages of base, mvtnorm and dunn.test (R Core Team. 2012) [22].

## Results

### Effect of Water on Survival and Development of Diapausing Eggs under WLD Conditions

**Fig 2:**
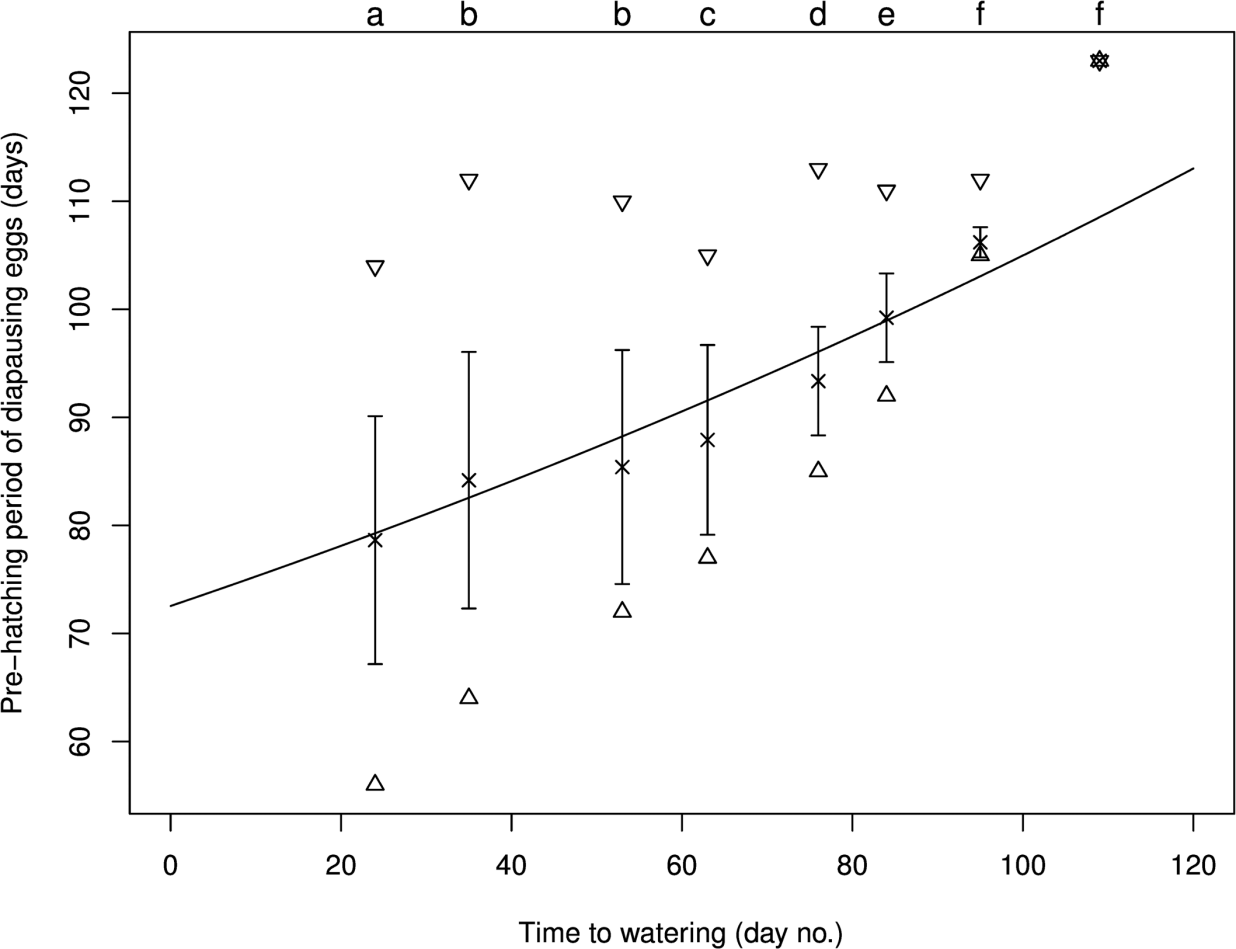
Relationship between pre-hatching period (*P*) of diapausing eggs and time to watering (*T*_*1*_) under warm long day conditions. The triangle (Δ), inverted triangle, the cross (×) and bar (┬) indicate the minimum, maximum, mean and standard deviation of the pre-hatching period in each treatment, respectively. The time to watering (*T*_*1*_) was 24, 35, 53, 63, 76, 84, 95 and 109 days, respectively. The exponential curve indicates the relationship (*P* = 72.53 exp (0.0037 *T*_*1*_)) between the pre-hatching period and time to watering.

When diapausing eggs of *A*. *lucorum* were kept consecutively under WLD conditions, diapause eventually terminated and the nymphs hatched, but the diapause termination rate and pre-hatching period were influenced by watering regimes (Table 1, Fig 2). The diapause termination rate was close to zero in diapausing eggs that were never watered (Table 1, Treatment A, only one nymph hatched out of 360 diapausing eggs) and in those that were not watered until day 109 of incubation (Table 1, Treatment I, only two nymphs hatched out of 336 diapausing eggs). In addition, the pre-hatching period of the diapause/post-diapause stage for both treatments (Table 1, Treatments A and I) was significantly longer than that for other treatments except for the treatment with watering once on day 95 of incubation (Table 1, Treatment H). These results indicated that water is crucial for the survival and diapause/post-diapause development of diapausing eggs of *A*. *lucorum*. The rate of diapause termination was higher than 70% for other water treatments and differed significantly between these treatments (Table 1, Treatments except for A and I). However, it had no simple relationship with water amount, watering time, watering frequency, or soaking frequency (Table 1). The pre-hatching period tended to be shorter with more frequent or earlier watering (Table 1, Fig 2). The pre-hatching period (*P*) exponentially increased with increasing time to watering or number of dry days (*T*_*1*_), and the relationship could be described as *P* = 72.53 exp (0.0037 *T*_*1*_) (Fig 2). This equation suggests that the pre-hatching period of diapausing *A*. *lucorum* eggs would be 72.53 ± 0.52 (S. E.) days in the situation where the water need was satisfied under WLD conditions. This prediction was consistent with the observed 72.76 days under the treatment of spraying with 2 ml of distilled water every 5 days (Table 1). Interestingly, the variation in the pre-hatching period tended to be smaller with the increased time to watering (Fig 2). Moreover, results also showed that excessive moisture (watering more frequently or in higher amounts) (Table 1, Treatments J, P, U and X) was detrimental to diapause/post-diapause development, e.g. the treatments of spraying with 2 ml of distilled water every 3 days (Table 1, Treatment J) and 3 ml of distilled water every 5 days (Table 1, Treatment P) resulted in a lower diapause termination rate and a longer pre-hatching period in comparison with the optimal treatment of spraying with 2 ml of distilled water every 5 days (Table 1, Treatment K).

### Effect of Water during Diapause Termination (Chilling)

**Fig 3:**
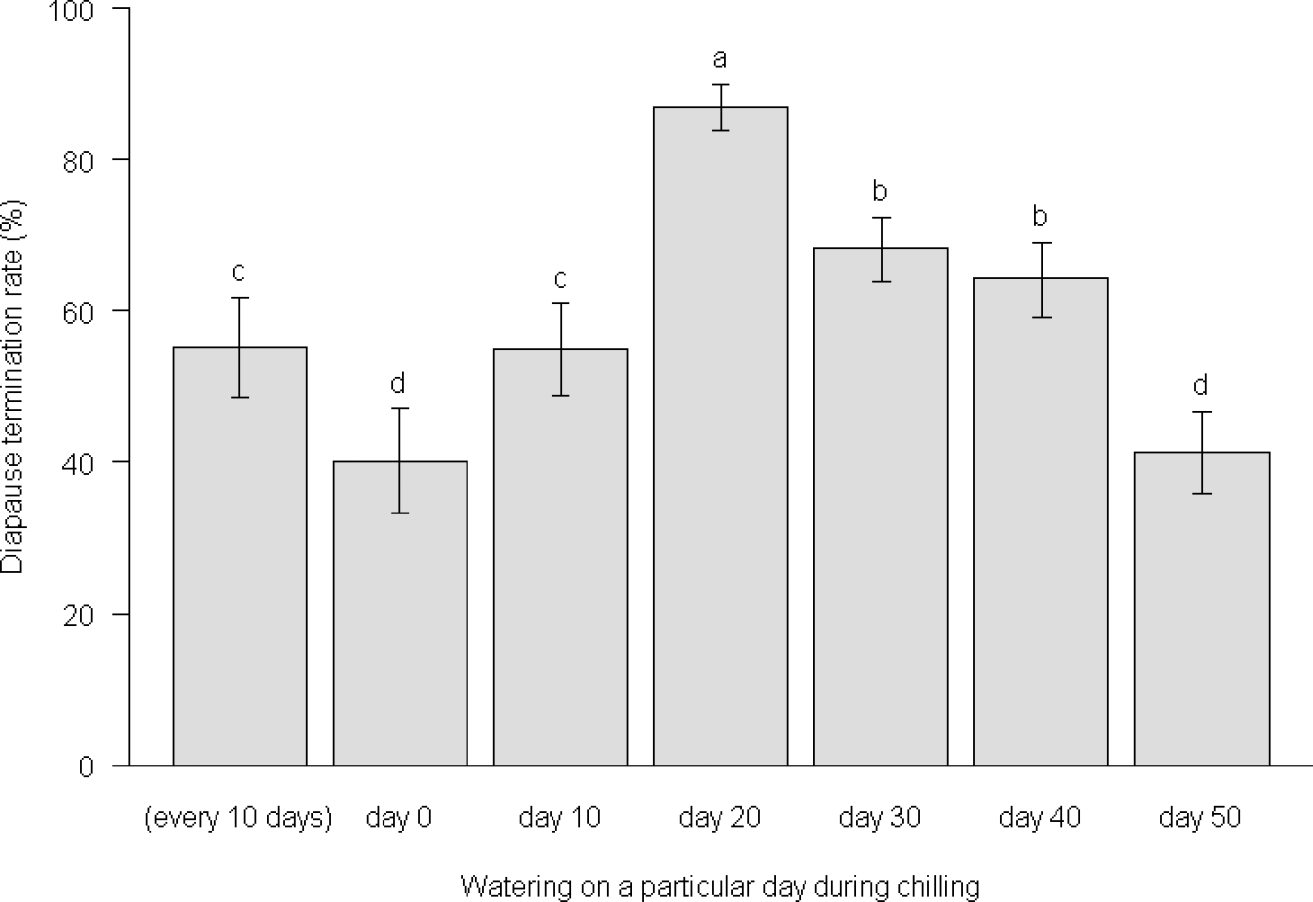
Diapause termination rate of diapausing *A*. *lucorum* eggs exposed to different water treatments during chilling at 4°C. The different lowercase letters indicated significant differences at the probability level of 0.05.

**Fig 4:**
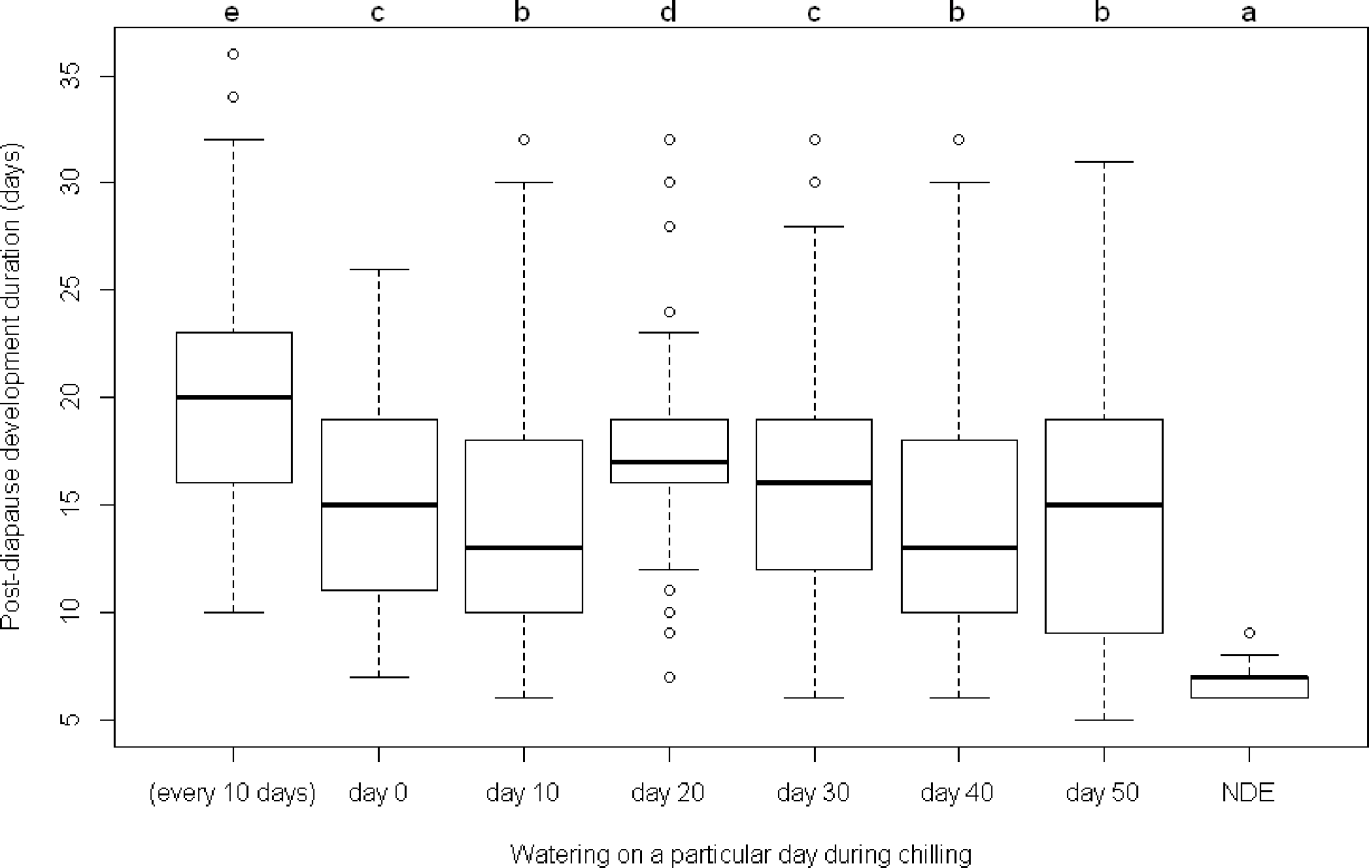
The post-diapause development duration of *A*. *lucorum* eggs with different water treatments during chilling at 4°C. NDE represents the developmental duration of non-diapause eggs. The different lowercase letters above each box indicated significant differences at the probability level of 0.05.

The diapause termination rate was highest in the treatment with watering on day 20 of chilling, and decreased for treatments with watering either earlier or later, when the diapausing eggs were watered only once during the diapause termination stage (Fig 3). Post-diapause development duration for this treatment was also the longest among the ‘watering once’ treatments (Fig 4). Watering more frequently, i.e., watering every 10 days, produced a moderate diapause termination rate but a longer post-diapause development duration compared with treatments of watering only once (Fig 3, Fig 4). Watering every 10 days had a similar effect on diapause termination as watering on day 10 of chilling judged by the termination rate, but the former significantly prolonged the post-diapause development duration. This finding suggested that excessive moisture or watering frequently was not beneficial for diapause development or termination. The post-diapause development duration of *A*. *lucorum* eggs was significantly longer for all water treatments during the diapause termination stage than the development duration of non-diapause eggs (Fig 4).

### Effect of Water during Post-Diapause Development

**Fig 5:**
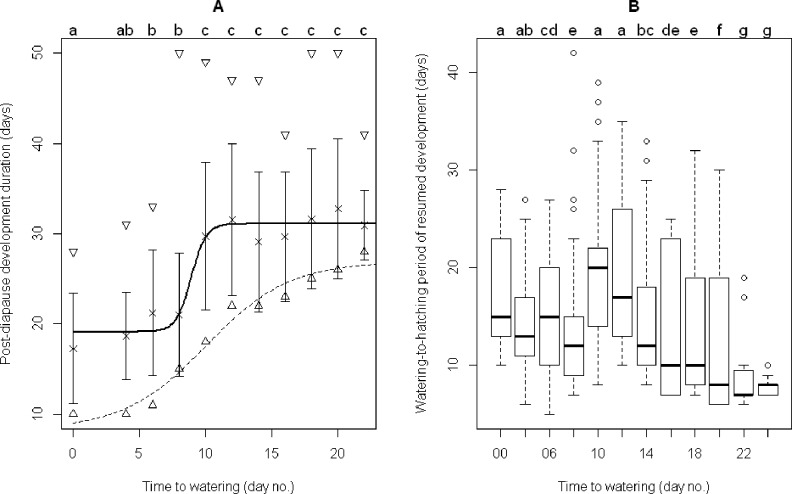
**The relationship between post-diapause development duration (A) and watering-to-hatching period of resumed development (B) and the time to watering (*T***_***2***_**) in post-diapause stage for *A*. *lucorum*.** The triangle (Δ), inverted triangle, the cross (×) and bar (┬) indicate the minimum, maximum, mean and standard deviation of development duration in each treatment, respectively. NDE represents the developmental duration of non-diapause eggs. The thick solid curve indicates the relationship between post-diapause development duration (*Dp*) and time to watering (*T*_*2*_) (dry days experienced) described by *Dp* = 19.12 + (31.08–19.12) / (1 + exp ((8.90—*T*_*2*_) / 0.56)). The thin dashed curve indicates the relationship between development duration of the post-diapause stage for the first hatchlings (*D*_*p1*_) and time to watering (*T*_*2*_) described by *D*_*p1*_ = 8.32 + (26.91–8.32) / (1 + exp ((10.07—*T*_*2*_) / 3.13)). The lowercase letters indicate significant differences at the probability level of 0.05.

Water uptake was very important in post-diapause development in *A*. *lucorum*. The development duration of post-diapause eggs *Dp* (defined as the duration from transferring to WLD conditions after chilling up to the hatching of nymphs) increased logistically with increased time to watering *T*_*2*_ (defined as the period from transferring to WLD conditions after chilling up to the watering day, or number of dry days experienced before eggs were watered during the post-diapause development duration), i.e., the nonlinear relationship could be described as: *Dp* = *A* + (*B—A*) / (1 + exp ((*xmid*—*T*_*2*_) / *scal*)), where parameters *A* = 19.12 ± 0.43 (t = 44.296; P < 0.0001), *B* = 31.08±0.36 (t = 85.189; P < 0.0001), *xmid* = 8.90±0.21 (t = 43.23; P < 0.0001) and *scal* = 0.56 ± 0.12 (t = 4.896, P < 0.0001) (Fig 5A, the thick solid curve). When the developmental duration only accounted for the first hatchlings, those parameters were *A* = 8.32 ± 1.76 (t = 4.73; P = 0.002), *B* = 26.91 ± 1.23 (t = 21.81; P < 0.0001), *xmid* = 10.07 ± 0.86 (t = 11.67; P < 0.0001), and *scal* = 3.13 ± 0.88 (t = 3.57; P = 0.009) (Fig 5A, the thin dashed curve). This suggested that the mean development duration of post-diapause eggs could be as short as 19.12 ± 0.43 days with the first nymph hatched within 8.32 ± 1.76 days. The eggs were separated into two groups based on the development duration of the post-diapause stage. The first group was composed of post-diapause eggs which had experienced 8 or less dry days and the second group was composed of eggs which had experienced 10 or more dry days (Fig 5A). Within each group, the post-diapause development duration was similar, but it was about 10 days longer in the second group than in the first group. This suggested that the post-diapause eggs might remain quiescent and not resume development until being watered. However, the watering-to-hatching period of resumed development for post-diapause eggs was obviously longer than that for the non-diapause eggs (Fig 5B). This suggested that post-diapause eggs did not develop in the same way as non-diapause eggs do. Moreover, within each group, the watering-to-hatching period of resumed development decreased with the increase of time to watering or number of dry days experienced (Fig 5B). This suggested that post-diapause development might be composed of two consecutive development periods and in each period the watering-to-hatching period of resumed development was negatively related to the length of dry days experienced. Thus, the effect of watering on post-diapause development was complex.

**Fig 6:**
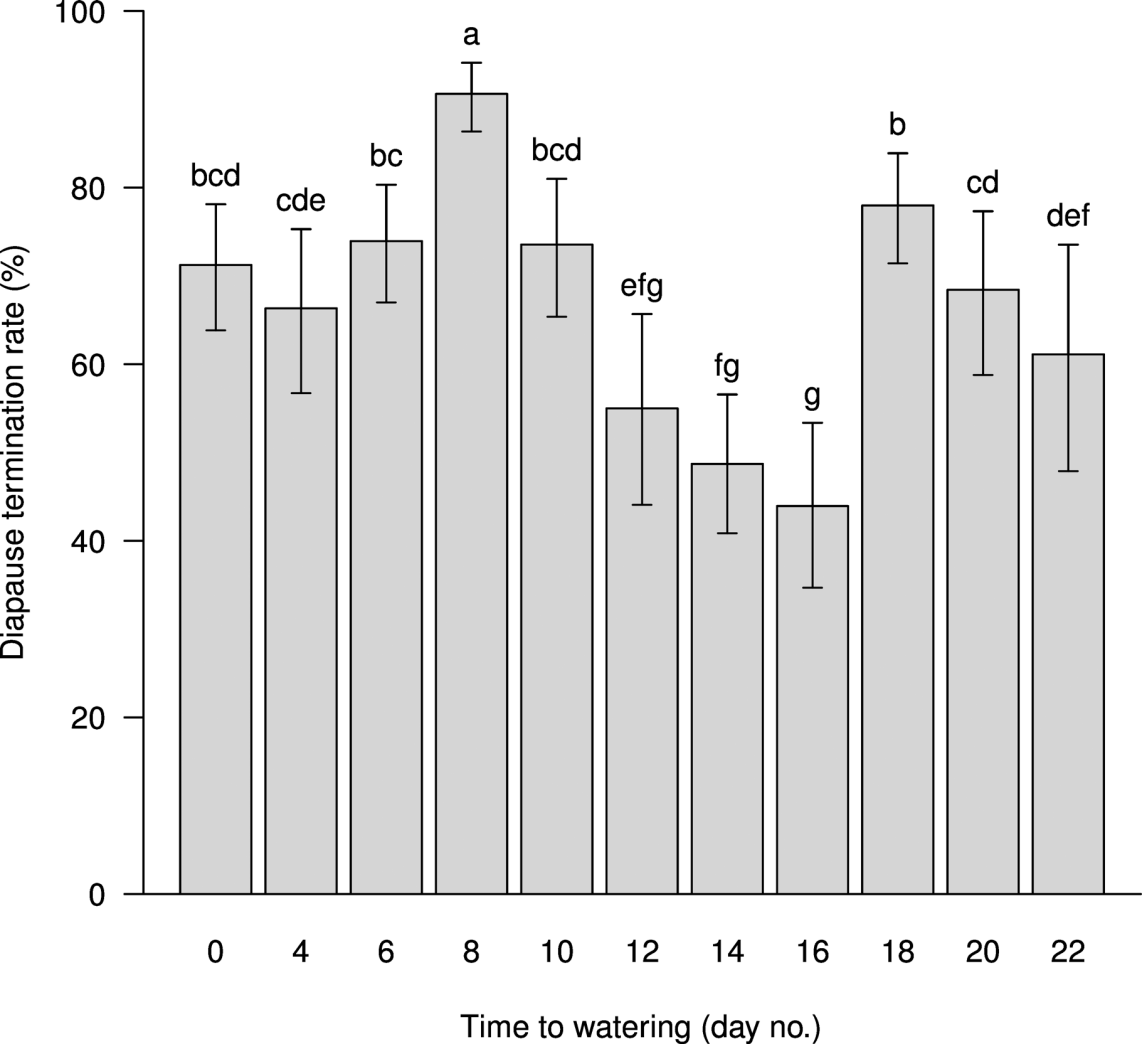
Diapause termination rate of *A*. *lucorum* eggs being watered at different times during the post-diapause stage. The lowercase letters indicate significant differences at the probability level of 0.05.

Time to watering (*T*_*2*_) (or in other words, number of dry days experienced before eggs were watered), at the post-diapause stage also influenced the diapause termination rate (Fig 6). The diapause termination rate varied with the number of dry days with the highest rate occurring for post-diapause eggs experiencing 8 dry days, and the second highest rate for eggs experiencing 18 dry days.

## Discussion

The present study focused on the influence of water on diapause development of *A*. *lucorum* not only for the whole diapause process, i.e., the diapause/post-diapause stage, but also more clearly for two sub-phases, i.e., diapause termination and post-diapause stages, using eggs with laboratory-induced diapause. The results indicated that water was crucial for diapause development of *A*. *lucorum* and diapausing eggs could not develop without water, which is consistent with earlier findings [6, 10]. In addition, we also found that a limited moisture regime was beneficial to diapause development of this species, but excessive moisture was detrimental to diapause development, as indicated by a decreased diapause termination rate and a prolonged developmental duration. A similar phenomenon has also been observed in the chrysomelid beetle *Homichloda barkeri*. In this species, moisture is critical for diapause termination and a sequence of wetting events followed by periods of dryness produced a high hatch rate [23]. This implied that both too dry and too humid winter and spring weathers would suppress survival and postpone hatching of overwintered *A*. *lucorum* eggs. This might help to explain why this pest is causing severe damage only in northern China [3]. The humid winter and spring in southern China and dry weather in western China might have suppressed *A*. *lucorum* populations and prevented serious damage in the south and west.

Water is one of the most important factors influencing life processes and has a complex influence on the whole diapause process in this species. The newly laid *A*. *lucorum* diapause-induced egg absorbs water and grows during 24 hours after oviposition [16] in order to initiate or prepare for diapause. Once the embryo enters diapause, i.e., in the diapause maintenance stage, the diapause egg stops absorbing water and growing [16]. However, the present study indicated that the diapausing egg might need to absorb water to terminate diapause and the number of dry days had complex effects on the diapause termination rate and the development duration. Our results on the watering of post-diapause eggs clearly showed that water was a trigger factor for *A*. *lucorum* to resume the development of eggs in the post-diapause quiescence phase, as also demonstrated in the false melon beetle *Atrachya menetriesi* and the corn rootworm *Diabrotica virgifera* [24, 25]. It was also observed that, with the increase in the number of dry days the eggs experienced in the diapause/post-diapause stage, the hatching period became narrower under WLD conditions (Fig 2). Therefore, water is not only a trigger factor but also an important factor regulating the process of diapause, and this is worth further investigation.

Low temperature plays an important role in the termination of winter diapause [17, 18]. Chilling is often effective in the termination of diapause under laboratory conditions [17, 18, 26, 27, 28] and this was demonstrated again in the present study for diapause termination of *A*. *lucorum* (Fig 3, Fig 6). In addition, the present study also found that egg diapause of *A*. *lucorum* could terminate spontaneously under WLD conditions and that the termination rate was generally greater than 80% (Table 1), which was higher than in most chilling treatments (Fig 3, Fig 6). If the lower termination rate represents a higher intensity of diapause, our results suggest chilling might have strengthened the intensity of diapause for those unhatched eggs. The proportion of unhatched eggs was relatively high, and could reach 60% in some chilling treatments. These unhatched eggs were plump (as opposed to shriveled) orange yellow and lacked any sign of red compound eyes, i.e. these eggs were still in diapause. Whether these eggs with prolonged diapause could survive for several years like the bean blister beetle *Epicauta gorhami* [27], chestnut weevil *Curculio sikkimensi*s [29] and wheat midge *Sitodiplosis mosellana* [30] merits further investigations. In the present study, we observed that the watering-to-hatching period of diapause eggs during post-diapause development duration was longer and more diverse than that of non-diapause eggs (Fig 4, Fig 5B). This implied that the watering-to-hatching period of resumed development in diapause eggs was different from the direct development of non-diapause eggs. Such qualitative differences have been found in other organisms [15]. Thus, it might not be appropriate to forecast the hatching date of overwintered eggs using a developmental threshold and estimated degree days for non-diapause eggs of *A*. *lucorum* [31]; rather, it seems necessary to investigate the threshold and degree days for the development of post-diapause eggs.
